# *N*,*N*′-Dicyclo­pentyl-*N*′′,*N*′′-dimethyl­phospho­ric triamide

**DOI:** 10.1107/S1600536811048549

**Published:** 2011-11-23

**Authors:** Akbar Raissi Shabari, Mehrdad Pourayoubi, Farnaz Ghoreishi, Banafsheh Vahdani

**Affiliations:** a Faculty of Chemistry, North Tehran Branch, Islamic Azad University, Tehran, Iran; b Department of Chemistry, Ferdowsi University of Mashhad, Mashhad, Iran

## Abstract

The P atom in the title mol­ecule, C_12_H_26_N_3_OP, has a distorted tetra­hedral configuration: its bond angles lie in the range 101.1 (2)–119.1 (2)°. The P—N bonds to the two cyclo­pentyl­amido moieties are significantly different [1.619 (4) and 1.643 (4) Å], with the shorter bond related to an *anti* orientation of the  lone electron pair of the corresponding N atom relative to the P=O bond. The O atom of the P=O group acts as a double hydrogen-bond acceptor and is involved in two different inter­molecular N—H⋯O(P) hydrogen bonds, building *R*_2_^2^(8) rings that are further linked into chains along [001].

## Related literature

For background to phospho­ric triamide compounds, see: Pourayoubi & Tarahhomi *et al.* (2011 ▶). For applications of phospho­ric triamides as oxygen-donor ligands, see: Pourayoubi & Golen *et al.* (2011 ▶). For bond lengths and angles in compounds having a [(N)P(O)(N)_2_] skeleton, see: Sabbaghi *et al.* (2011 ▶). For double hydrogen-bond acceptors, see: Steiner (2002 ▶).
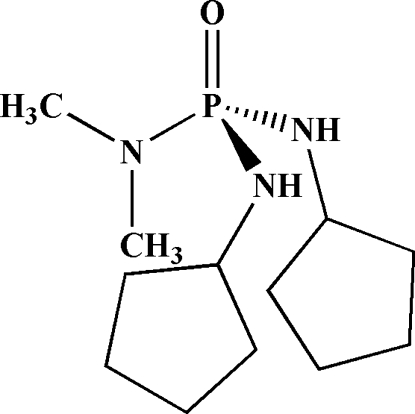


## Experimental

### 

#### Crystal data

C_12_H_26_N_3_OP
*M* *_r_* = 259.33
Orthorhombic, 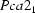

*a* = 10.962 (5) Å
*b* = 16.663 (5) Å
*c* = 8.079 (5) Å
*V* = 1475.7 (12) Å^3^
*Z* = 4
Mo *K*α radiation
μ = 0.18 mm^−1^
*T* = 291 K
0.35 × 0.11 × 0.05 mm

#### Data collection

Stoe IPDS 2T Image Plate diffractometer
Absorption correction: multi-scan [*MULABS* (Blessing, 1995 ▶) and *PLATON* (Spek, 2009 ▶)] *T*_min_ = 0.961, *T*_max_ = 1.000
7303 measured reflections
2573 independent reflections
1482 reflections with *I* > 2σ(*I*)
*R*_int_ = 0.095

#### Refinement

*R*[*F* ^2^ > 2σ(*F* ^2^)] = 0.056
*wR*(*F* ^2^) = 0.108
*S* = 0.88
2573 reflections
151 parameters
1 restraint
H-atom parameters constrained
Δρ_max_ = 0.16 e Å^−3^
Δρ_min_ = −0.23 e Å^−3^
Absolute structure: Flack (1983 ▶), 1093 Friedel pairs
Flack parameter: −0.20 (18)

### 

Data collection: *X-AREA* (Stoe & Cie, 2009 ▶); cell refinement: *X-AREA*; data reduction: *X-AREA*; program(s) used to solve structure: *SHELXTL* (Sheldrick, 2008 ▶); program(s) used to refine structure: *SHELXTL*; molecular graphics: *SHELXTL*; software used to prepare material for publication: *SHELXTL*, *PLATON* (Spek, 2009 ▶) and *enCIFer* (Allen *et al.*, 2004 ▶).

## Supplementary Material

Crystal structure: contains datablock(s) I, global. DOI: 10.1107/S1600536811048549/ld2034sup1.cif

Structure factors: contains datablock(s) I. DOI: 10.1107/S1600536811048549/ld2034Isup2.hkl

Additional supplementary materials:  crystallographic information; 3D view; checkCIF report

## Figures and Tables

**Table 1:** Hydrogen-bond geometry (Å, °)

*D*—H⋯*A*	*D*—H	H⋯*A*	*D*⋯*A*	*D*—H⋯*A*
N1—H1⋯O1^i^	0.85	2.13	2.960 (5)	167
N2—H2⋯O1^ii^	0.85	2.33	3.131 (5)	158

Symmetry codes: (i) 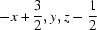; (ii) 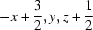.

**Fig. 1.**
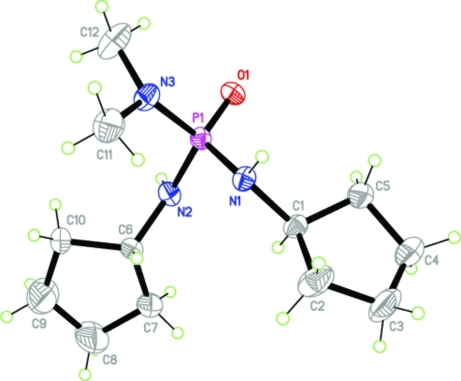
The molecular structure of the title compound with ellipsoids shown at the 50% probability level.

**Fig. 2.**
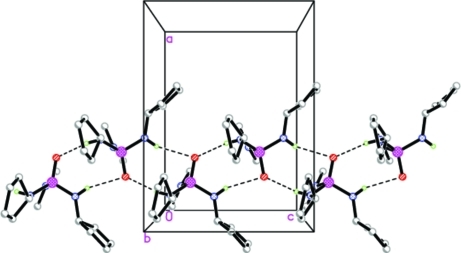
A view of the crystal packing showing the formation of 1-D arrangement through N—H···O hydrogen bonds (shown as dashed lines). Carbon bound H atoms have been omitted for clarity.

## supplementary crystallographic information

### Comment

The structure determination of the title molecule was done as part of a project on the synthesis of new phosphoric triamide compounds (Pourayoubi & Tarahhomi *et al.*, 2011) and their application as oxygen donor ligands (Pourayoubi & Golen *et al.*, 2011).

The P═O and P—N bond lengths and the C—N—P bond angles match those found for the other compounds having a [(N)P(O)(N)_2_] skeleton (Sabbaghi *et al.*, 2011).

The tetrahedral configuration of phosphorus atom (Fig. 1) is significantly distorted as it has also been noted for other phosphoric triamides: the bond angles at the P atom vary in the range from 101.1 (2) [N1—P1—N3] to 119.1 (2)° [O1—P1—N1].

The O atom of the P═O group acts as a double hydrogen-bond acceptor (Steiner, 2002); so, in the crystal structure, each molecule is hydrogen-bonded to two adjacent molecules by forming the [N—H]_2_···O(P) grouping within a 1-D hydrogen-bonded arrangement parallel to the *c* axis (Fig. 2, Table 1).

### Experimental

Synthesis of ((CH_3_)_2_N)P(O)Cl_2_: [(CH_3_)_2_NH_2_]Cl (0.184 mol) and P(O)Cl_3_ (0.552 mol) were refluxed for 8 h and afterwards the excess of P(O)Cl_3_ was removed in vacuum.

Synthesis of title compound: a solution of cyclopentylamine (14.8 mmol) in CH_3_CN (25 ml) was added to a solution of ((CH_3_)_2_N)P(O)Cl_2_ (3.7 mmol) in CH_3_CN (15 ml) at 273 K. After stirring for 4 h, the solvent was removed and the product was washed with deionized water and recrystallized from CH_3_CN at room temperature.

### Refinement

The N-bound H atoms were found in difference Fourier map and then constrained to refine with the parent atoms with *U*_iso_(H) equal to 1.2*U*_eq_(N). The remaining H atoms were positioned geometrically and constrained to refine in a riding-model approximation with *U*_iso_(H) = 1.2*U*_eq_(C), or 1.5*U*_eq_(methyl). A rotating group model was applied to the methyl groups.

### Crystal data

**Table:**

C_12_H_26_N_3_OP	*F*(000) = 568
*M**_r_* = 259.33	*D*_x_ = 1.167 Mg m^−^^3^
Orthorhombic, *P**c**a*2_1_	Mo *K*α radiation, λ = 0.71069 Å
Hall symbol: P 2c -2ac	Cell parameters from 2536 reflections
*a* = 10.962 (5) Å	θ = 2.0–27.5°
*b* = 16.663 (5) Å	µ = 0.18 mm^−^^1^
*c* = 8.079 (5) Å	*T* = 291 K
*V* = 1475.7 (12) Å^3^	Needle, colourless
*Z* = 4	0.35 × 0.11 × 0.05 mm

### Data collection

**Table:**

Stoe IPDS 2T Image Plate diffractometer	2573 independent reflections
Radiation source: fine-focus sealed tube	1482 reflections with *I* > 2σ(*I*)
graphite	*R*_int_ = 0.095
Detector resolution: 0.15 pixels mm^-1^	θ_max_ = 25.5°, θ_min_ = 2.2°
ω scans	*h* = −11→13
Absorption correction: multi-scan [*MULABS* (Blessing, 1995) and *PLATON* (Spek, 2009)]	*k* = −20→20
*T*_min_ = 0.961, *T*_max_ = 1.000	*l* = −8→9
7303 measured reflections	

### Refinement

**Table:**

Refinement on *F*^2^	Secondary atom site location: difference Fourier map
Least-squares matrix: full	Hydrogen site location: inferred from neighbouring sites
*R*[*F*^2^ > 2σ(*F*^2^)] = 0.056	H-atom parameters constrained
*wR*(*F*^2^) = 0.108	*w* = 1/[σ^2^(*F*_o_^2^) + (0.0401*P*)^2^] where *P* = (*F*_o_^2^ + 2*F*_c_^2^)/3
*S* = 0.88	(Δ/σ)_max_ < 0.001
2573 reflections	Δρ_max_ = 0.16 e Å^−^^3^
151 parameters	Δρ_min_ = −0.23 e Å^−^^3^
1 restraint	Absolute structure: Flack (1983), 1093 Friedel pairs
Primary atom site location: structure-invariant direct methods	Flack parameter: −0.20 (18)

### Special details

**Table:**

Experimental. IR (KBr, cm^-1^): 3290, 3151, 2955, 2866, 2835, 2794, 1459, 1291, 1197, 1159, 1107, 1090, 1030, 993, 932, 889, 762, 703, 555, 496, 464.
Geometry. All e.s.d.'s (except the e.s.d. in the dihedral angle between two l.s. planes) are estimated using the full covariance matrix. The cell e.s.d.'s are taken into account individually in the estimation of e.s.d.'s in distances, angles and torsion angles; correlations between e.s.d.'s in cell parameters are only used when they are defined by crystal symmetry. An approximate (isotropic) treatment of cell e.s.d.'s is used for estimating e.s.d.'s involving l.s. planes.
Refinement. Refinement of *F*^2^ against ALL reflections. The weighted *R*-factor *wR* and goodness of fit *S* are based on *F*^2^, conventional *R*-factors *R* are based on *F*, with *F* set to zero for negative *F*^2^. The threshold expression of *F*^2^ > σ(*F*^2^) is used only for calculating *R*-factors(gt) *etc*. and is not relevant to the choice of reflections for refinement. *R*-factors based on *F*^2^ are statistically about twice as large as those based on *F*, and *R*- factors based on ALL data will be even larger.

### Fractional atomic coordinates and isotropic or equivalent isotropic displacement parameters (Å^2^)

**Table:**

	*x*	*y*	*z*	*U*_iso_*/*U*_eq_	
P1	0.83134 (10)	1.79029 (6)	0.70933 (17)	0.0402 (3)	
O1	0.6995 (2)	1.78019 (14)	0.7413 (4)	0.0476 (9)	
N1	0.8963 (4)	1.73464 (16)	0.5707 (5)	0.0418 (10)	
H1	0.8771	1.7422	0.4700	0.050*	
N2	0.9056 (3)	1.77094 (19)	0.8814 (5)	0.0452 (10)	
H2	0.8616	1.7816	0.9651	0.054*	
N3	0.8558 (4)	1.8809 (2)	0.6328 (4)	0.0545 (12)	
C1	0.9062 (4)	1.6462 (2)	0.5936 (6)	0.0458 (12)	
H1A	0.9222	1.6352	0.7108	0.055*	
C2	1.0064 (5)	1.6094 (3)	0.4926 (9)	0.088 (2)	
H2A	1.0841	1.6147	0.5489	0.106*	
H2B	1.0121	1.6354	0.3854	0.106*	
C3	0.9725 (6)	1.5223 (3)	0.4730 (10)	0.100 (2)	
H3A	0.9884	1.5046	0.3606	0.121*	
H3B	1.0201	1.4894	0.5481	0.121*	
C4	0.8410 (6)	1.5149 (3)	0.5115 (10)	0.096 (2)	
H4A	0.8291	1.4821	0.6093	0.115*	
H4B	0.7979	1.4903	0.4197	0.115*	
C5	0.7943 (5)	1.5994 (2)	0.5411 (7)	0.0678 (17)	
H5A	0.7594	1.6216	0.4408	0.081*	
H5B	0.7328	1.5999	0.6275	0.081*	
C6	1.0370 (4)	1.7749 (2)	0.9067 (6)	0.0480 (12)	
H6A	1.0773	1.7747	0.7984	0.058*	
C7	1.0855 (5)	1.7061 (3)	1.0069 (7)	0.0700 (15)	
H7A	1.0208	1.6825	1.0729	0.084*	
H7B	1.1188	1.6650	0.9348	0.084*	
C8	1.1810 (7)	1.7386 (4)	1.1141 (11)	0.139 (2)	
H8A	1.1771	1.7136	1.2224	0.167*	
H8B	1.2607	1.7279	1.0669	0.167*	
C9	1.1626 (7)	1.8212 (4)	1.1282 (9)	0.139 (2)	
H9A	1.2401	1.8488	1.1166	0.167*	
H9B	1.1302	1.8334	1.2371	0.167*	
C10	1.0791 (5)	1.8495 (2)	1.0034 (8)	0.0702 (17)	
H10A	1.1195	1.8872	0.9301	0.084*	
H10B	1.0099	1.8761	1.0544	0.084*	
C11	0.9700 (5)	1.9066 (3)	0.5626 (8)	0.0808 (19)	
H11A	0.9552	1.9478	0.4820	0.121*	
H11B	1.0094	1.8619	0.5103	0.121*	
H11C	1.0215	1.9274	0.6487	0.121*	
C12	0.7793 (6)	1.9460 (2)	0.6916 (10)	0.093 (2)	
H12A	0.7768	1.9877	0.6098	0.139*	
H12B	0.8122	1.9669	0.7929	0.139*	
H12C	0.6982	1.9263	0.7109	0.139*	

### Atomic displacement parameters (Å^2^)

**Table:**

	*U*^11^	*U*^22^	*U*^33^	*U*^12^	*U*^13^	*U*^23^
P1	0.0424 (6)	0.0398 (4)	0.0385 (6)	−0.0033 (5)	−0.0014 (9)	0.0013 (7)
O1	0.0381 (18)	0.0585 (16)	0.046 (3)	−0.0026 (13)	−0.0026 (18)	0.0068 (16)
N1	0.054 (3)	0.0364 (17)	0.035 (2)	−0.0031 (17)	0.000 (2)	0.0055 (17)
N2	0.039 (3)	0.057 (2)	0.039 (2)	−0.0055 (19)	0.006 (2)	−0.0082 (19)
N3	0.060 (3)	0.0419 (19)	0.061 (3)	−0.0004 (19)	0.010 (2)	0.0020 (16)
C1	0.058 (3)	0.042 (2)	0.037 (3)	0.010 (2)	−0.002 (3)	0.001 (2)
C2	0.073 (4)	0.060 (3)	0.131 (6)	0.006 (3)	0.038 (5)	0.004 (4)
C3	0.112 (6)	0.067 (4)	0.122 (6)	0.022 (3)	0.016 (6)	−0.035 (4)
C4	0.098 (5)	0.051 (3)	0.140 (7)	0.007 (3)	−0.010 (6)	−0.012 (3)
C5	0.057 (4)	0.052 (3)	0.095 (5)	−0.002 (2)	−0.006 (3)	−0.002 (3)
C6	0.038 (3)	0.050 (3)	0.056 (3)	0.000 (2)	−0.004 (3)	0.001 (2)
C7	0.068 (4)	0.058 (3)	0.085 (4)	0.005 (3)	−0.017 (4)	0.007 (3)
C8	0.158 (6)	0.107 (3)	0.154 (5)	0.012 (4)	−0.095 (5)	−0.004 (3)
C9	0.158 (6)	0.107 (3)	0.154 (5)	0.012 (4)	−0.095 (5)	−0.004 (3)
C10	0.064 (4)	0.047 (3)	0.099 (5)	−0.007 (2)	−0.017 (4)	−0.016 (3)
C11	0.086 (5)	0.059 (3)	0.097 (5)	−0.016 (3)	0.005 (4)	0.025 (3)
C12	0.129 (5)	0.052 (3)	0.098 (6)	0.015 (3)	0.034 (6)	−0.001 (4)

### Geometric parameters (Å, °)

**Table:**

P1—O1	1.478 (3)	C5—H5A	0.9700
P1—N1	1.619 (4)	C5—H5B	0.9700
P1—N2	1.643 (4)	C6—C7	1.500 (6)
P1—N3	1.653 (4)	C6—C10	1.539 (6)
N1—C1	1.489 (4)	C6—H6A	0.9800
N1—H1	0.8499	C7—C8	1.463 (8)
N2—C6	1.456 (5)	C7—H7A	0.9700
N2—H2	0.8489	C7—H7B	0.9700
N3—C11	1.440 (6)	C8—C9	1.395 (8)
N3—C12	1.451 (6)	C8—H8A	0.9700
C1—C2	1.499 (7)	C8—H8B	0.9700
C1—C5	1.515 (6)	C9—C10	1.441 (7)
C1—H1A	0.9800	C9—H9A	0.9700
C2—C3	1.508 (6)	C9—H9B	0.9700
C2—H2A	0.9700	C10—H10A	0.9700
C2—H2B	0.9700	C10—H10B	0.9700
C3—C4	1.480 (8)	C11—H11A	0.9600
C3—H3A	0.9700	C11—H11B	0.9600
C3—H3B	0.9700	C11—H11C	0.9600
C4—C5	1.516 (6)	C12—H12A	0.9600
C4—H4A	0.9700	C12—H12B	0.9600
C4—H4B	0.9700	C12—H12C	0.9600
			
O1—P1—N1	119.06 (19)	H5A—C5—H5B	108.9
O1—P1—N2	108.3 (2)	N2—C6—C7	113.0 (4)
N1—P1—N2	104.78 (19)	N2—C6—C10	113.9 (4)
O1—P1—N3	109.13 (18)	C7—C6—C10	103.7 (4)
N1—P1—N3	101.13 (19)	N2—C6—H6A	108.7
N2—P1—N3	114.55 (18)	C7—C6—H6A	108.7
C1—N1—P1	120.9 (3)	C10—C6—H6A	108.7
C1—N1—H1	106.5	C8—C7—C6	106.9 (4)
P1—N1—H1	117.9	C8—C7—H7A	110.3
C6—N2—P1	126.8 (3)	C6—C7—H7A	110.3
C6—N2—H2	116.2	C8—C7—H7B	110.3
P1—N2—H2	110.6	C6—C7—H7B	110.3
C11—N3—C12	114.1 (4)	H7A—C7—H7B	108.6
C11—N3—P1	124.1 (3)	C9—C8—C7	108.1 (6)
C12—N3—P1	117.8 (3)	C9—C8—H8A	110.1
N1—C1—C2	113.0 (4)	C7—C8—H8A	110.1
N1—C1—C5	114.6 (4)	C9—C8—H8B	110.1
C2—C1—C5	103.3 (4)	C7—C8—H8B	110.1
N1—C1—H1A	108.6	H8A—C8—H8B	108.4
C2—C1—H1A	108.6	C8—C9—C10	110.9 (6)
C5—C1—H1A	108.6	C8—C9—H9A	109.5
C1—C2—C3	105.7 (4)	C10—C9—H9A	109.5
C1—C2—H2A	110.6	C8—C9—H9B	109.5
C3—C2—H2A	110.6	C10—C9—H9B	109.5
C1—C2—H2B	110.6	H9A—C9—H9B	108.0
C3—C2—H2B	110.6	C9—C10—C6	106.4 (4)
H2A—C2—H2B	108.7	C9—C10—H10A	110.5
C4—C3—C2	107.3 (4)	C6—C10—H10A	110.5
C4—C3—H3A	110.3	C9—C10—H10B	110.5
C2—C3—H3A	110.3	C6—C10—H10B	110.5
C4—C3—H3B	110.3	H10A—C10—H10B	108.6
C2—C3—H3B	110.3	N3—C11—H11A	109.5
H3A—C3—H3B	108.5	N3—C11—H11B	109.5
C3—C4—C5	106.6 (4)	H11A—C11—H11B	109.5
C3—C4—H4A	110.4	N3—C11—H11C	109.5
C5—C4—H4A	110.4	H11A—C11—H11C	109.5
C3—C4—H4B	110.4	H11B—C11—H11C	109.5
C5—C4—H4B	110.4	N3—C12—H12A	109.5
H4A—C4—H4B	108.6	N3—C12—H12B	109.5
C1—C5—C4	104.4 (4)	H12A—C12—H12B	109.5
C1—C5—H5A	110.9	N3—C12—H12C	109.5
C4—C5—H5A	110.9	H12A—C12—H12C	109.5
C1—C5—H5B	110.9	H12B—C12—H12C	109.5
C4—C5—H5B	110.9		
			
O1—P1—N1—C1	−65.4 (4)	C5—C1—C2—C3	−32.8 (6)
N2—P1—N1—C1	55.8 (4)	C1—C2—C3—C4	17.9 (8)
N3—P1—N1—C1	175.1 (3)	C2—C3—C4—C5	4.3 (8)
O1—P1—N2—C6	−178.9 (3)	N1—C1—C5—C4	158.6 (5)
N1—P1—N2—C6	53.0 (4)	C2—C1—C5—C4	35.2 (6)
N3—P1—N2—C6	−56.8 (4)	C3—C4—C5—C1	−24.6 (7)
O1—P1—N3—C11	−169.5 (4)	P1—N2—C6—C7	−138.0 (4)
N1—P1—N3—C11	−43.2 (4)	P1—N2—C6—C10	104.0 (5)
N2—P1—N3—C11	68.9 (4)	N2—C6—C7—C8	−142.0 (5)
O1—P1—N3—C12	34.3 (5)	C10—C6—C7—C8	−18.2 (6)
N1—P1—N3—C12	160.6 (4)	C6—C7—C8—C9	20.8 (8)
N2—P1—N3—C12	−87.3 (4)	C7—C8—C9—C10	−14.8 (9)
P1—N1—C1—C2	−158.0 (4)	C8—C9—C10—C6	2.8 (8)
P1—N1—C1—C5	84.1 (5)	N2—C6—C10—C9	132.9 (5)
N1—C1—C2—C3	−157.2 (5)	C7—C6—C10—C9	9.7 (6)

### Hydrogen-bond geometry (Å, °)

**Table:**

*D*—H···*A*	*D*—H	H···*A*	*D*···*A*	*D*—H···*A*
N1—H1···O1^i^	0.85	2.13	2.960 (5)	167
N2—H2···O1^ii^	0.85	2.33	3.131 (5)	158
C11—H11B···N1	0.96	2.50	2.978 (6)	110
C12—H12C···O1	0.96	2.45	2.926 (5)	111

Symmetry codes: (i) −*x*+3/2, *y*, *z*−1/2; (ii) −*x*+3/2, *y*, *z*+1/2.
